# Shark New Antigen Receptor (IgNAR): Structure, Characteristics and Potential Biomedical Applications

**DOI:** 10.3390/cells10051140

**Published:** 2021-05-08

**Authors:** Salma Nassor Juma, Xiaoxia Gong, Sujie Hu, Zhengbing Lv, Jianzhong Shao, Lili Liu, Guiqian Chen

**Affiliations:** 1 College of Life Science and Medicine, Zhejiang Sci-Tech University, Hangzhou 310018, China; salmanassor60@yahoo.com (S.N.J.); 17826819184@163.com (X.G.); husj5322@163.com (S.H.); zhengbingl@zstu.edu.cn (Z.L.); 2 Zhejiang Provincial Key Laboratory of Silkworm Bioreactor and Biomedicine, Hangzhou 310018, China; 3 College of Life Science, Zhejiang University, Hangzhou 310058, China; shaojz@zju.edu.cn

**Keywords:** IgNAR, shark, antibody, therapeutic application

## Abstract

Shark is a cartilaginous fish that produces new antigen receptor (IgNAR) antibodies. This antibody is identified with a similar human heavy chain but dissimilar sequences. The variable domain (VNAR) of IgNAR is stable and small in size, these features are desirable for drug discovery. Previous study results revealed the effectiveness of VNAR as a single molecule or a combination molecule to treat diseases both in vivo and in vitro with promising clinical applications. We showed the first evidence of IgNAR alternative splicing from spotted bamboo shark (*Chiloscyllium plagiosum*), broadening our understanding of the IgNARs characteristics. In this review, we summarize the discoveries on IgNAR with a focus on its advantages for therapeutic development based on its peculiar biochemistry and molecular structure. Proper applications of IgNAR will provide a novel avenue to understand its special presence in cartilaginous fishes as well as designing a number of drugs for undefeated diseases.

## 1. Introduction

**Figure 1:**
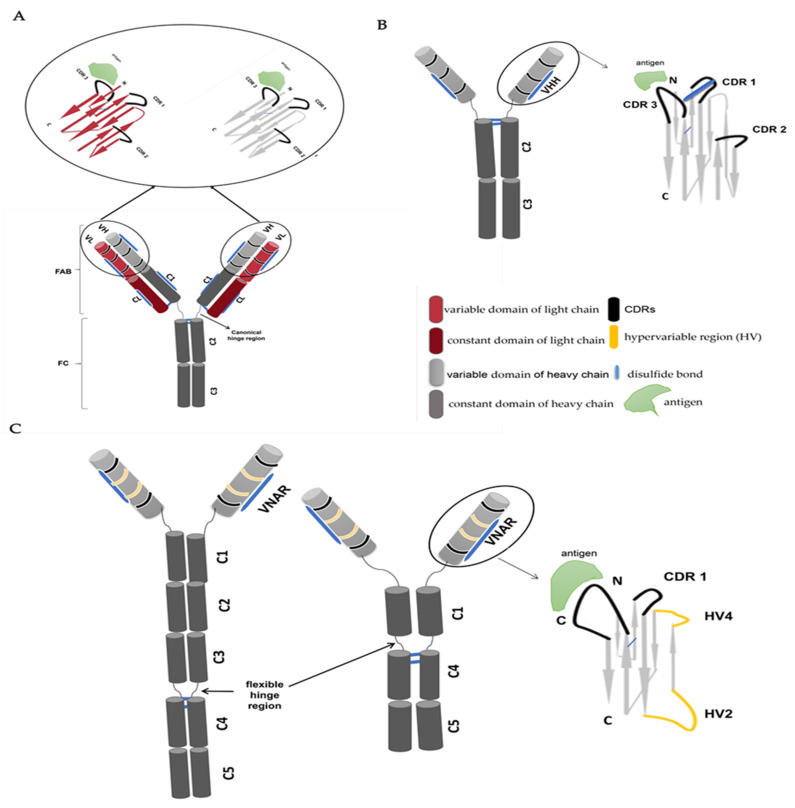
Structure of the antibodies. (**A**) IgG has two identical heavy chains and two identical light chains. (**B**) camelids’ antibodies contain only a heavy chain without the C1 domain. (**C**) shark antibody-containing only heavy chains with hypervariable regions 2 and 4, and a flexible hinge region between C3 and C4. Alternative spliced form of IgNAR in bamboo sharks with only C1, C4, and C5 domains; the variable domain matched the complete form of IgNAR with an extra disulfide bond between CDR1 and CDR3 and HV2 and HV4 chains with the antigen in the long CDR3 as shown in VHH.

Most conventional antibodies (IgG) are heterodimers (Figure 1A) with two heavy chains (VHs) and two light chains (VLs) [1]. Structurally, IgGs are distributed into antigen-binding fragment (Fab) and Fragment-crystallizable (Fc) portion. Fab portion contains one constant domain of the heavy chain (C1) and one constant domain of the light chain (CL), as well as one variable domain of heavy chain (VH) and one variable domain of light chain (VL). The variable domain of each chain is responsible for antigen interactions due to the existence of paratope (the antigen-binding site) [1,2]. The variable domains of IgG are linked by a flexible peptide into an antiparallel sheet (Figure 1A). Each variable domain has three loops of complementarity-determining regions (CDRs). The disulfide bond in the variable domain between the framework region one (FR1) and the framework region three (FR3) is formed by a canonical cysteine residue [3]. Fc portion [4] contains two constant domains (C2 and C3) of the heavy chain for their biological activities [5]. The characteristic flexibility of the IgG is characterized by the hinge region in the middle part of the heavy chain [6].

The existence of an antibody with only a heavy chain was first reported from camelids in 1993 [7]. Two years later, a publication announced that sharks possess this type of antibody [8]. Sharks belong to cartilaginous fish and this fish identified with isotype IgM, IgW, and IgNAR [9], however, a constant domain (C1–C5) of IgNAR are identified to be closely related to primordial IgW isotype [10].

The variable domain of camelids and sharks identified with molecular weights of approximately 15 kDa and 12 kDa that are referred to as VHH and VNAR, respectively (Figure 1C); the antigen-binding site is formed in only one single domain of the heavy chain [1]. Inter-chain disulfide bonds joint the structure of VHH and VNAR domain [11,12,13]. The camelid antibody consists of the constant domain two (C2) and constant domain three (C3) with a hinge region between the variable region and the constant domain two (C2), and constant domain one (C1) is absent due to a donor splice site mutation [1] (Figure 1B). The shark antibody (Figure 1C) consists of C1–C5 domains and lacks canonical hinge regions between the variable region and the constant domains; the structural flexibility of IgNAR is caused by the linkage of a disulfide bridge to the constant domain (C3–C4) [14]. In this review, we will include our interesting recent discovery of spliced form from white-spotted bamboo sharks (*Chiloscyllium plagiosum*) that attained its flexible hinge region between constant domain (C1) and constants domain (C4), where the flexible hinge region structure in nurse sharks identified between constant domain (C3) and constant domain (C4) [15]. The variable regions in CDRs of different sharks vary due to the presence of extra cysteines, which is used to classify VNARs as the common method to date. With the reference to amino acid sequences, the sizes of the CDR3 of VH are shorter compared to those of VHH and VNAR [11]. VNAR was discovered to be sharing structural homology with immunoglobulin light chain and T-cell receptor variable regions [16,17].

## 2. Characteristic of Variable of IgNAR

Different shark species [18], including the banded wobbegong (Orectolobus maculatus), spiny (Squalus acanthias), bamboo (Chiloscyllium plagiosum), and nurse (Ginglymostoma cir-ratum) sharks produce different VNARs [15,18]. Variable domains of sharks are formed by four hypervariable loops: CDR1 and CDR3, somatic mutations result in the deletion of CDR2 [14], this position of CDR2 replaced by very short strand referred to as HV2 [19]. The HV4 is sited between HV2 and CDR3, this HV4 is believed to contribute to antigen binding [14,20]. 8 β-strands form the antigen-binding site of VNAR instead of 10 as in mammalian variable domains, this making VNAR being smallest (12 kDa) antigen-binding domain known in the vertebra to date [16,21].

**Figure 2:**
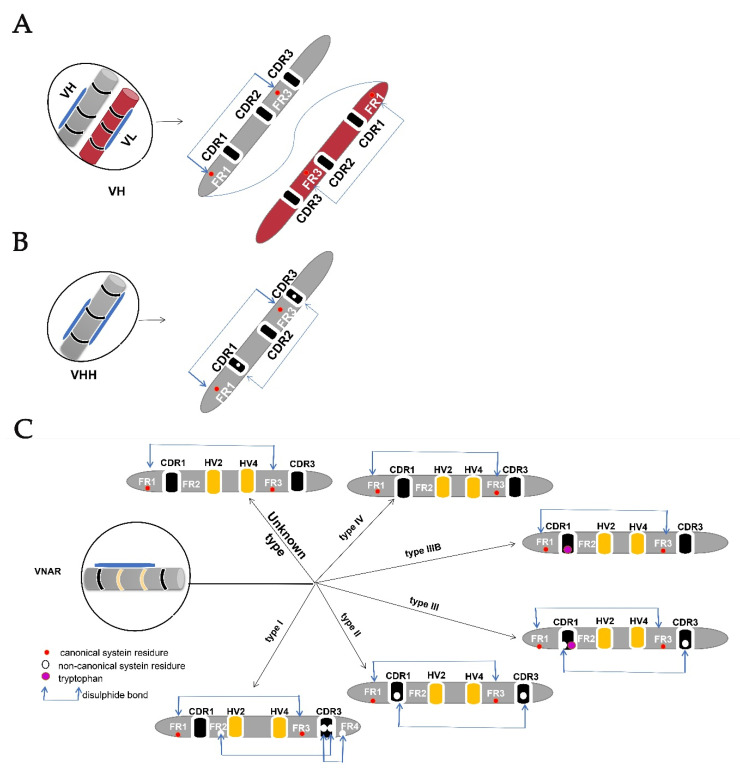
Variable domain structure. (**A**) IgG antibody variable domain (VH) with a disulfide bond connection be-tween the variable domain and two conserved canonical cysteine residues in the framework region (FR1) and (FR3). (**B**) HCAbs variable domain (VHH) with an extra cysteine residue forming a disulfide bond between CDR1 and CDR3 despite the conserved canonical cysteine residues. (**C**) Variable domain (VNAR) of IgNAR lacking a CDR2, with vari-ous numbers of noncanonical cysteine residues that give rise to different types of VNAR and hypervariable regions (HV2 and HV4).

Two canonical cysteine residues hold two beta-sheets in the framework regions (FRs) 1 and framework regions (FRs) 3. In addition to these canonical cysteines, CDRs may have non-canonical/extra cysteine residues that form additional disulfide bonds within the variable domain [22]. These features differentiate VH from the VHH and VNAR domains (Figure 2), and to date are used as a means of classifying VNAR based on the presence or absence of these extra cysteine residues within the hypervariable region [5,11,12,14,15,18,23]. VNAR is classified into four types (I, II, III, and IV), the subtypes are further divided based on the number of additional cysteines contained [18]. However, there are discovered IgNARs that did not fit any of these described types due to the VNAR domain mutation rate [22].

The type I variable domains of IgNAR had been reported only in nurse sharks (Gin-glymostoma cirratum) [12,14], but have now been also identified in the wobbegong shark (Orectolobus ornatus) [18]. VNAR type 1 has non-canonical cysteine residues in FR2 and FR4, and two paired residues in CDR3 [14].

The non-canonical cysteine residues in CDR3 form disulfide bonds with non-canonical cysteine residues within the FR2 and FR4 [16]. Type II VNAR forms intramolecular disulfide bonds between CDRI and CDR3 due to the presence of an additional cysteine residue. Types II VNARs have finger-like CDR3s that can bind into pockets or grooves, for example, active site clefts of enzymes [14]. Type III and type II VNAR is similar, possessing non-canonical cysteine residue in CDR1 and CDR3 [5]. These types of VNARs were hypothesized to fight against pathogens during the developmental stages of sharks but further developed to be matured classes of VNAR after maturation, which provides a more expansive immune repertoire [12,23]. Type IV VNAR lacks non-canonical disulfide bonds but contains only two cysteine residues that hold VNAR together [11]. This provides flexibility to the antigen-binding site of type IV VNAR [12,14]. Another structurally different VNAR type named type IIIb has been reported as a type IV VNAR with a tryptophan residue in CDR1 like the one in type III VNAR [5,14], lacking non-canonical cysteine residues (and consequently non-canonical disulfide bonds). Some VNARs cannot be characterized in any of the four known types [22], which were isolated from different shark species and displayed good binding abilities to antigens [18]. Therefore, more researches are required to reveal these VNARs with peculiar cysteine arrangement that fall in neither of the four known types.

VNARs types can be significantly different among various species of sharks [22], while some reported sharks such as small-spotted catsharks, banded hound sharks, and the wobbegong sharks contained a novel variant without non-canonical cysteine residue [24]. Current studies revealed the existence of various VNARs with unknown types caused by the inconsistency of cysteine numbers as well as locations [22], while some studies revealed the therapeutic advantages of unknown VNAR types of different shark species [18]. The criteria used for VNAR classification should be reviewed because previous studies were mainly in nurse sharks that possess type 1 other shark species are not. Therefore, advancements in classifying VNAR using various methods are important to miss out on VNAR that give out immune responses. 

An unknown type of VNAR has been identified. A large phage library was constructed from six adult nurse sharks (*Ginglymosto macirratum*) for diversification of the VNAR library because previous studies showed types I VNAR are possessed by nurse sharks. Canonical cysteines located at 21 and 82 amino acid were used as the main criterion to characterize type I–IV VNAR. Eventually, around 5% of the total VNARs did not fit into any known types, while 30% of the VNAR possess various numbers of cysteines with neither type I nor type II behavior [22]. In our recent discovery, the presence of two canonical cysteines located at 22 and 83 amino acid were used as a means of classification of type I-IV VNARs. Approximately, 0.3% of the total VNARs did not match any of the four known VNARs types [15]. 

Another unknown VNAR type was identified by randomization of CDR3 from banded hound sharks (*Triakis scyllium)*. This VNAR displayed the therapeutic potential. Hen egg-white lysozyme gives successful isolation of antigen-specific IgNAR variable region without immunization of target antigen. Amino acid sequences of CDR3 had either one or two cysteine residues (behavior of type I-IV VNAR), but this VNAR was contradictory to previously reported results from (*Ginglymostoma cirratum*) nurse shark [25]. Again, Zielonka et al. isolated VNAR of unknown types. This VNAR domain was successfully developed using yeast surface display from a CDR3-randomized of bamboo shark (*Chiloscyllium plagiosum*), showing high-affinity characteristics [19]. Therefore, various means of classification are recommended as some studies produce VNARs with therapeutic advantages but nowhere to be found among the VNARs types.

Categorization of IgNAR on reviewing variable domain only seems to be not sufficient due to the higher variability in the CDRs regions [24]. The new method has proposed the possibility of using the C1 domain sequence to identify IgNAR clusters, and this is because the constant domain (C1) showed distinctiveness to other constant domains (C2-C5) of IgW. The peculiarity of C1 appeared to be an important factor to be considered in characterizing IgNAR clusters [24]. 

Compared to the other four constant domains, the C1 domain revealed higher stability with the good antigen-binding ability to its variable region [15], thus the C1 domain plays a significant role in structural alterations that increase affinity against a specific antigen [10]. A flexible (non-canonical) linker between VNAR and C1 domain gives out the dimerization that produces a wide angle of VNAR and binding to multiple epitopes [14]. A unique form of C1 domain was identified from the study of characterization of complete IgNAR heavy chain constant domain of brown-banded bamboo sharks (*Chiloscyllium punctatum*) as well as evolutionary relationship determination with different species. This IgNAR was discovered with the presence of two distinct IgNAR types designated as (IgNAR-1 CH type and the IgNAR-2 CH type) and 13 unique C1 sequences [24]. The results of the study were related to our recent discovery in white-spotted bamboo (*Chiloscyllium plagiosum*) sharks [15]. We identified two types of C1 domains, one with a short α-helix and the other without a short α-helix. C1 with a short α-helix hypothesized to have higher stability than the other. Based on these findings, it suggests using constants domain (C1) sequences for comparing novel clusters of IgNAR types in future studies on cartilaginous fish [24].

## 3. Alternatives Spliced Constant Domain of IgNAR

The constant region of shark species is less studied, but recent evidence revealed a primary role in stability maintenance of the antibody [24]. Shark constant domains are identical to those in IgG’s constant domains except for their unstructured loop; however, these unconventional IgNAR domains are very stable compared to the domains of conventional antibodies. IgNARs can maintain biological activities in shark blood containing 350 mmol/L urea and 1000 mOsm/kg of osmotic salt ions [26]. 

Our recent discovery [15] revealed a splicing alternative form in the white-spotted bamboo (*Chiloscyllium plagiosum*) shark. Surprisingly, only C1, C4, and C5 domains were present during the cloning of the complete IgNAR sequence form with 5 constant domains, but C2 and C3 were absent. We designated this spliced sequence as IgNAR_short_ ΔC2-C3 (Figure 1C). According to phylogenetic analysis, the identified spliced sequences of white-spotted bamboo (*Chiloscyllium plagiosum*) belonged to the IgNAR family and closely related to the brown-banded bamboo shark (*Chiloscyllium punctatum*). VNAR of spliced form might have the antigen-specific binding ability as a structural model prediction was consistent with the structural characteristics of IgNAR. Conserved tryptophan and cysteine residues were also present which could be involved in the formation of disulfide bonds and structural folds. The interchain disulfide bond between C1 and C4 is predicted to be the result of an unpaired cysteine in the flexible hinge region of IgNARshort (ΔC2-C3) [15]. Therefore, we speculated the flexibility attained in spliced form between C1-C4 is due to an unpaired cysteine contained. This result is consistent with “Matthias J. Feige” and colleague study, the stalk of IgNAR flexibility is maintained by a disulfide bond between C3 and C4 caused by unpaired cysteine residue [10]. With references to previous studies as well as our bamboo shark results [15], IgNAR full sequences of shark species needed to be examined to diverse our knowledge of this antibody.

## 4. VNAR Complex Structure Responding to Different Proteins

The structural elements of the IgNAR constant domain could also influence the folding pathway of the antibody which increases the stability. Thermostability of variable region of IgNAR isolated from different sharks species are superior to that of conventional antibodies [27]. Immunization of a horn shark (*Heterodontus francisci*) with recombinant human tumor necrosis factor-alpha rhTNFα produced VNAR with higher thermostability behavior due to its ability to identify rhTNFα and neutralize it in vitro [28]. This attribute makes it a suitable candidate as a therapeutic antibody, mainly due to the ability to transplant the stable structural motif into other domains.

Inhibition of full T cell activation can be attained by blocking the interaction with inducible costimulator (ICOS) through Induced costimulatory ligand (ICOSL). The study of Marina Kovaleva1 and colleagues revealed the reduction of inflammation to a murine model of non-infectious uveitis by inhibiting this ICOSL using specific VNARs that recognized human ICOSL isolated from an immunized nurse shark. The anti-mouse ICOSL VNAR Fc that was constructed showed a high affinity for inducible T-cell co-stimulator ligand (ICOSL) and high penetration of the cornea in a mouse model of uveitis. Results of their experiments demonstrated the efficiency and potentiality of the VNAR binding domain for the treatment of auto-inflammatory conditions [29]. Obinna C. Ubah and colleagues designed VNAR fusion anti-hTNF-α Quad-X™ that showed improvement in potency over Humira^®^ [30]. These findings from previous studies identified the usefulness of the VNAR complex to the protein that is a good target for those diseases. 

However, we should keep in mind the other side of the previous findings, despite the immune responses of the isolated VNAR to bind with complex structure and responding to different proteins, but not all VNARs of shark species were tested to produce an adaptive immune response [26]. Crouch et al. used human serum albumin (HSA) and (HEL) as antigens to immunize spotted catsharks (*Scyliorhinus canicula*), after 37 weeks of the immunization process, they did not find evidence of antigen-specific VNAR [31]. 

## 5. Therapeutic Biomedical Applications of IgNAR

### Production of Shark IgNAR

IgNARs can be produced either by immunized, non-immunized [32,33,34] or semi-synthetic library form [35,36]. The desired gene of the shark antibody can be inserted into bacteria after insertion into a vector as a transporting agent. Displaying technology can either be yeast, ribosome, or phage [32] followed by selection and analysis. In our laboratory, we are immunizing sharks and isolate VNARs from the phage library technique introduced [37]. In this approach, antibody-antigen binding is displayed by inserting antibody DNA into a vector and introducing phenotype and genotype into phage genomes, together with the construction of a large library for the particular antibody of interest and higher affinity to antigen [38], to produce VNAR antibody from immunized shark [37,39]. 

**Figure 3:**
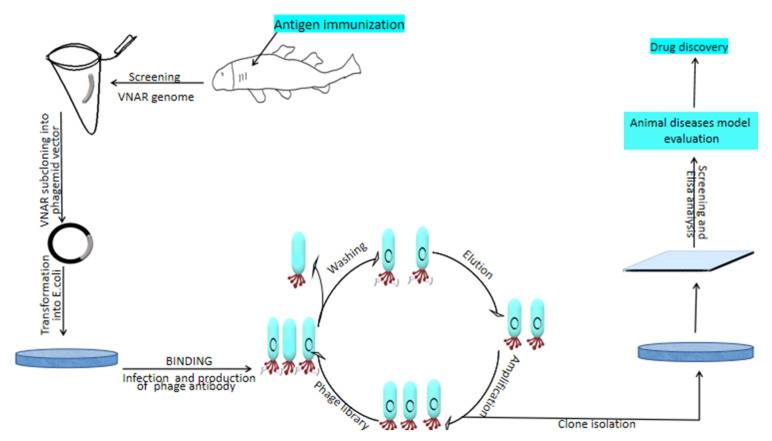
IgNAR production using a phage display library. Specific antigens are used to immunize sharks and induce an immune response about 3–6 months and then subcloned VNAR into the phagemid vector for phage antibody display assays. The identified antibodies after the screening can be used to evaluate their efficacy in vitro and in vivo.

Once the antigen proteins are ready, they will be injected into sharks to induce an immune response (Figure 3). After several months, blood samples and shark spleen are taken for transcriptome and mass spectrum analyses. Phagemid vector can be used to carry specific antibody fragments to transform *E. coli* cells and generate a library. The genome encoding an antibody protein of interest derived from the phage coding protein genes leads to the display of proteins on the outside of bacterial cells (showing the phenotype of interest) carrying the genes in their genome (showing the genotype of interest). Only antibodies from the phage library that fit and bind to the antigen are recovered while the others are washed away. Then, the selection of clones followed by screening using an ELISA binding assay to identify antibody-antigen specificity [38]. After the screening, the identified IgNARs are further evaluated for efficacy using animal disease models to test their in vivo functions (Figure 3).

## 6. Immunoglobulin VNAR in Drug Discovery

Different companies and academic institutions have been considering using VNAR shark libraries to identify potential therapeutics for human diseases [18]. The unique features of VNAR may be useful for drug discovery; the isolated VNAR called V13 produced using phage display to select against a human recombinant vascular endothelial growth factor (VEGF165) cytokine was isolated from an immunized *Heterodontus francisci* shark. They founded this V13 VNAR penetrated the cornea without the need for an injection and without causing ocular surface abrasions or signs of discomfort in an animal model (probably due to its small size and long CDR3 of 27 amino acids). Their findings demonstrated the potential applicability of VNAR V13 as a new drug candidate for vascular eye diseases [40].

Another VNAR produced by immunizing nurse shark with human-induced costimulatory ligand (ICOSL), reformatted by using fragments crystallization (Fc) region of a human antibody. When anti-mouse ICOSL Fc introduced to uveitis mouse model of (IRBP) inter-photoreceptor retinoid-binding protein-induced uveitis tested in vivo, eventually decreases the inflammation compared to untreated mouse [18,29].

Other findings showed the functional activity of isolated VNAR using a semi-synthetic phage display library bounded to a panel of BAFF (B cell-activating factor) with low nM potency. All receptors (BR3, TACI, and BCMA) selected in the study blocked by anti-BAFF VNARs, suggesting that the bio-specific antibodies with added functionality may be effective in treating complex autoimmune diseases [41].

Arthritis (tenderness and swelling joints) has become a serious human disease in elderly populations, especially in women. Tumor necrosis factor-alpha (TNF-α) is a cytokine involved in systemic inflammation [12,42] and is identified to be a good target to treat arthritis [13]. The structure of anti-TNF-α VNAR-human TNF-α complexes has been solved and novel epitopes have been discovered by crystallization analysis where VNAR interacts with two adjacent TNF-α protomers [13,30]. Another VNAR isolated from sharks has shown promising features to alleviate the progress of arthritis in animal models, and average in vivo arthritis inhibition and histopathology scores are 88% and 86% using Quad-XTM at doses of 0.5 and 30 mg/kg, respectively [13], demonstrating the valuable application of IgNAR in drug discovery again.

## 7. Viability of VNAR in Relation with Immunogenicity

Therapeutic antibodies in humans could potentially cause anti-drug antibodies (ADAs) that lead to unfavorable outcomes of the medication [43]. The desired immunogenicity in humans is exhibited by lowering the differences between the natural IgG and bispecific antibody format [17,43,44,45]. The engineered full-length mAbs give rise to reduction into single-chain Fv fragments (scFvs) that retain the binding specificity of the parent antibody [46,47,48], thus leading to low immunogenicity [49] as well as a potentially unique molecule to be used especially in cancer treatment [46].

Sharks possess low overall sequence homology (≈30%) to human VH/VL sequences [44], but the VNAR domain observed by crystallization data of their framework regions in a similar manner to human immunoglobulin variable domains, [17,44,50]. Thus, the similarities in the arrangement of framework regions are advantageous to therapeutic developments due to the direction to humanized VNAR binders. Here, undesired immunogenicity of shark antibody can be minimized via humanizing VNAR by replacing amino acid residues in the framework of the VH domain [43] and utilized as a tiny molecule domain delivery vehicle. It was reported that VNAR can be combined with monospecific antibodies to develop bispecific agents [41]. Individual VNARs have been converted into VNAR-Fc fusions [29]. ScFv (single-chain fragment variable) antibodies have been constructed mainly from B lymphocytes in humans [46].

The tightly packed VNAR allows penetration into the active sites of different targets. VNAR antibody is desirable in the drug development field due to its small peculiarity. The combined molecules of VNAR with other proteins were reported to be effective to imitate adaptations of the parental VNAR without losing its efficiency. The stability of the VNAR domain through its disulfide bond can significantly improve the stability of human antibodies when transferred to the variable region of those [14].

The first VNAR designated as E06 was isolated from immunization of dogfish (*Squalus acanthias*) shark species, based on binding epitopes recognized by VNAR binds specifically and with high affinity to humans, this study did not base on determining anti-drug antibodies (ADAs,) but involving in non-human primates (NHP) but showed no evidence of anti-VNAR antibody production [51]. Later, scientists humanized VNAR by converting more than half of their CDRs to those of a human germline V_k_1 sequence, DPK9 [17]. The specificity and affinity of antigen-binding of the parental VNAR (E06) were retained by humanized VNAR (huE06 v1.1) upon binding with human serum albumin (HSA) [17]. Therefore, this study gives a foundation for further design and humanization of shark IgNARs [17]. John Steven and colleagues extended the study of E06 VNAR by using humanized VNAR (huE06v1.10) as a template to isolate domains with improved biophysical properties and reduced antigenicity [50]. In their study, the immunogenicity of lead clones was assessed in a T-cell proliferation assay using ProImmune Ltd. REVEAL^®^ Immunogenicity System DC–T cell assay. The prediction of immunogenicity in silico modeling of VNAR domains was lower, both the wild-type E06 and humanized variants had a very low response index (RI) compared to positive controls, thus a low level of immunogenicity with similar values for those seen for a human Fc region was predicted [50].

Cotton et al. reports about oncofetal protein receptor tyrosine kinase ROR1 that overexpressed on solid tumor. The cross-reactivity revealed by the constructed VNAR-drug conjugates targeted ROR1, thus even the highly similar family member (ROR2) could not bind. However, the study is in progress to assess the in vivo efficiency, but the complex structure of VNAR was successfully combined with sdAbs and scFv which were directed to other cell-surface protein targets [52].

Delta-like ligand 4 (DLL4) is prone to overgrowing tumors, which is a good target for pancreatic cancer. VNAR against Delta-like ligand 4 (DLL4) was conjugated to therapeutic nanoparticle (NPs) poly(lactic-co-glycolic) acid PEGylated by using surface maleimide functional groups. This study result also showed the specificity binding ability of these nanoconjugates [53].

Blood-brain barrier (BBB) penetration is a major challenge in therapeutic development. The large size of conventional antibodies is a restriction to this penetration [54], but the small size of VNAR is a good weapon, allowing them to reach the buried epitopes, facilitating the discovery of mouse–human cross-species reactive sDAbs. This is a feature not always accessible with conventional IgGs [54]. The study published in July 2017 by Frank S. Walsh revealed the penetration ability of VNAR fusion with IgG. The combination achieved by merged a single-chain variable fragment (scFv) of shark domain with terminus end (N- or C-) of the heavy and light chain of an IgG. The bispecific antibody formats produced retained antibody-dependent cell-mediated cytotoxicity [43,55].

The report from the previous study revealed the low inherent immunogenicity of the VNAR. Impact of anti-drug antibodies (ADAs) detected on preclinical in vivo efficacy using non-immunoglobulin VNAR fusion anti-hTNF-α biologics (Quad-X™ and D1-NDure™-C4) and Humira^®^, a brand of adalimumab [30], demonstrating the promising application of VNAR in biomedical industries.

## 8. IgVNAR Potential in Immunoassays

The isolation of single-domain antibodies has been successful using a naïve nurse shark VNAR library with PCR extension assembly and self-ligation (EASeL). Based on this technique, glypican3, human epidermal growth factor receptor 2 (HER2), and programmed cell death-1 (PD1) as well as the viral antigens middle east respiratory syndrome (MERS) and spike proteins [22] have been tested. Non-immunized adult spiny dogfish (*Squalus acanthias*) and smooth dogfish (*Mustelus canis*) sharks have also been used to construct libraries using Luminex100 and traditional ELISA assays. Shark VNAR sdAb libraries can be used to specifically demonstrate their binding ability to different antigens, as well as identifying agents that have been suggested as new venues to be used for pathogen detection [56].

The high thermal stability of the variable region of the receptor VNAR from sharks is important for the diagnosis of different diseases. Monoclonal antibodies in malaria rapid diagnostic tests (RDTs) using VNAR shark binders have been studied, with splenocytes used to construct a single domain antibody (sdAb). Three recombinant malaria biomarker proteins for *Plasmodium falciparum* (PfHRP2-histidine-rich protein 2, PfpLDH-plasmodium lactate dehydrogenase, and fructose 1, 6-biphosphate aldolase) from immunized wobbegong sharks (*Orectolobus ornatus*) were successfully used to isolate target-specific bacteriophage VNARs using phage display technology to identify antigen binders [57].

Consideration of the variable domain of sharks as therapeutic agents is caused by the peculiarity of this antibody’s structure from different discoveries: short α-helix structure of constant domain (C1) [15], salt bridge, and hydrophobic core are thought to contribute to the stability of constant domains [10]. Compared with murine mAbs and scFvs, shark VNAR sdAbs molecules are more sensitive and thermally stable to viral nucleoprotein (NP) of ZEBOV [58]. The variable domain of the shark antibody can be modified into a high number of formats and fused to various molecules and produced a satisfactory outcome. The advantage of VNAR stability in extreme pHs, for example, incubation with acid produced by gastric glands [23,59]. The two distinct clones of VNAR reported to have a good binding ability and stability to *P. gingivalis* KgP [58]. All these findings are important to consider IgNAR as a satisfactory therapeutic agent.

## 9. Strength of VNAR Domain over Traditional mAbs

The production of recombinant VNAR is easier due to no post-translational modifications are required and expressions of VNAR are performed well using *E. coli* than in mammalian cells [60], high solubility is achieved because the hydrophilic residues were presented within VNAR surfaces [61], therefore it is not expensive compared to the production of traditional mAbs [60,61]. After injection of a specific antigen, it takes around 4–6 months to get the desired antibody because the IgNAR response of sharks is slower than the process observed in mammals [5,61].

There are many residues in IgG which are not available in VNAR, and thus making it the smallest antibody fragment [48,61]. The long CDR3 gives the VNAR uniqueness to be able to target a small epitope that can be easily reached by conventional IgG. The binding-specific activity of VNAR can be attained even after exposure to temperature up to 95 °C [61]. Besides, VNAR exhibited high physicochemical stability [48]. This feature is desirable for applications of VNAR than traditional antibodies in a therapeutic and biotechnological setup. During the comparison between commercial reagents derived from conventional polyclonal sera and monovalent VNAR clone to P. falciparum AMA1, the data showed that heat stability of purified recombinant VNAR was superior to that of conventional mAbs, and even the refolding property of VNAR was retained when the temperature increased to 80 °C. Therefore, the usage of VNAR as a binder to malaria is preferable [62].

## 10. Conclusions

IgNAR’s structures endow the molecule with specific functions; for example, its small size and thermal stability are advantageous for penetration into the epitopes of tumors; these features have made IgNAR a focus for diverse applicable conditions. We summarized the progress on IgNAR discoveries as valuable conjugate and highlighted its structural features and potential applications for drug discovery with pathogen detection as well as immunogenicity to show the value of IgNAR as a biomedical agent. Despite the uniqueness of structural features of IgNAR and its characteristic in the broadening drugs target to undefeated diseases, extensive efforts are needed to overcome challenges in the time-consuming process of raising and immunizing sharks during the production of IgNAR and on the establishment of dynamic monitoring methods for IgNAR in sharks. More studies are still required to study the categories of IgNAR clusters in various ways than being consistent with extra cysteine residues only. Additionally, novel technologies should be considered to make sure that VNAR molecules are delivered to their targets at the right place in the body to treat diseases such as tumors (a task that remains challenging).

## Data Availability

All data is included in the manuscript.
